# Supplementary figures and images for: Evaluating the diagnostic and triage performance of digital and online symptom checkers for the presentation of myocardial infarction; A retrospective cross-sectional study
Source: PLOS Digit Health. 2024 Aug 5;3(8):e0000558. doi: 10.1371/journal.pdig.0000558 (PMC11299816; doi:10.1371/journal.pdig.0000558)

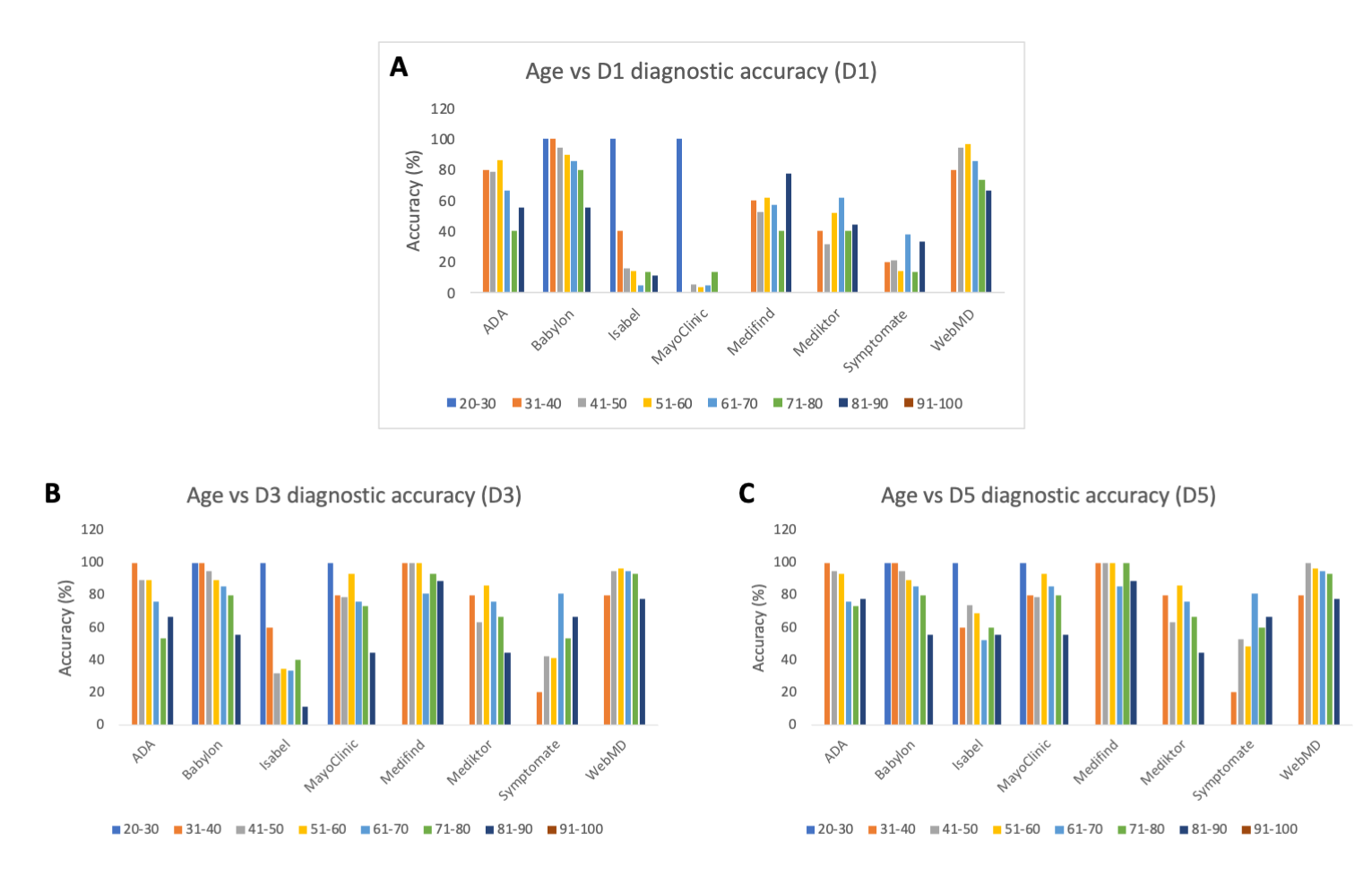

Supplement: S2 Fig — Symptom checker D1 (A), D3 (B) and D5 (C) performance for decade-wise age categories. (TIFF) [file pdig.0000558.s002.tiff]
