# Supplementary material for: Evaluating the diagnostic and triage performance of digital and online symptom checkers for the presentation of myocardial infarction; A retrospective cross-sectional study
Source: PLOS Digit Health. 2024 Aug 5;3(8):e0000558. doi: 10.1371/journal.pdig.0000558 (PMC11299816; doi:10.1371/journal.pdig.0000558)
Supplement: Attachment — Submitted filename: Wallace et al Review.pdf [file pdig.0000558.s003.pdf]

Review of Wallace et al Evaluating the Diagnostic and Triage Accuracy of Digital and Online Symptom Checkers for the Presentation of Myocardial Infarction; A Retrospective Cross-Sectional Study

Confidential

To the authors and editor,

Please find below my review of the Research Article noted above.

Please note that my primary expertise is regulatory science, so my comments will primarily relate to these themes and comments outside this expertise may be discarded if the editor and authors do not share my concerns.

Overall recommendation: **minor revision**

Thematic comments:

I highlight the following thematic points of feedback:

A. I would challenge the authors on two methodological issues. First, I don't get a good sense of what the human standard of care is for triaging or diagnosis of MI. Generally, a Clinical Evaluation Report would start by identifying the current standard of care and then assembling evidence against that bar. Nevertheless, the findings are still of grave concern, given the significant variance. Second, much of the discussion focuses on the diagnostic accuracy of symptom checkers. However, it would surprise me if most of these manufacturers and lay users really view the output as a 'diagnosis,' perhaps at best it's an indicative diagnosis and certainly information that allows for diagnosis but it strikes me that the triaging claim is a more appropriate standard to hold symptom checkers to, at least for now.y

B. The authors have anonymised the included symptom checkers. This impairs the ability for others to replicate these results and will diminish any safety activity that might be taken on the basis of the findings. I can perhaps guess why the symptom checkers have been anonymised but the decision will limit the impact of the paper.

Roche

C. It appears that there has been a significant period of time between an initial draft of this paper and the submission of this manuscript. It also appears that there has been some efforts to update some references. However, I would perhaps question two elements. First, making some assumptions about which symptom checkers have been included, I think some of the claims are out of date and now incorrect. For instance, assuming Ada Health is included, this device now has Class IIa authorisation under the EU Medical Device Regulation (since 2022). This would necessitate updating the numbers regarding the classification of symptom checkers. I suspect that I would not be the only one to notice this discrepancy. Second, the literature on how to assess the performance of symptom checkers has made some modest progress, which hasn't necessarily been captured, despite its relevance. For instance, Painter (2022) *Online symptom checkers: recommendations for a vignette-based clinical evaluation standard* speaks to some of the same issues identified in this article.

Notwithstanding the above, I think this is an article that adds to the existing literature and offers an important contribution.

Specific comments and suggested corrections:

**Line reference**	**Comment / suggested correction**
110-113	This line notes that FDA has recognised that apps can be recognised as software as a medical device. I find the wording of this sentence to be odd and potentially misleading for a number of reasons: a) the fact that the software in question is an app is largely irrelevant with respect to whether the app qualifies as a medical device or falls within the FDA's enforcement discretion, b) the 21st Century Cures Act actually restricts rather than expands what the FDA can qualify as a medical device. I'd recommend the sentence be written along the following lines: "Following International Medical Device Regulators Forum (IMDRF) Guidance [CITATION], software that has a medical purpose (as defined in the guidance) qualifies as a medical device and is regulated as such. Although, despite apparent international harmonisation, there is still heterogeneity between IMDRF members as to what software qualifies from jurisdiction to jurisdiction."
113-115	This sentence seems strange for two reasons: a) I believe the phrase should be 'NICE recommended' rather than 'NICE approved', b) there seems to be no citation here, despite this being a quantitative assessment, c) the statement in parentheses probably needs elaboration, i.e. "(nor do they state the classification where they do qualify as a medical device)", d) I'm not sure the sentence noted above necessarily leads to the next "... there is a dearth of evidence on their safe usage" I'd consider rephrasing to something like "... there is therefore a lack of regulatory requirements relating to safety such as post market surveillance requirements" as there can be evidence relating to safety generated outside of the regulated domain.
125-127	I would highly suggest here that the authors flag the discussion of symptom checker intended purpose

Confidential

	and use of disclaimers discussed later in the article, as many of these SCs specifically disclaim the ability to diagnose.
127-129	The authors should note that it is the manufacturer's legal responsibility to appropriately classify their device with rationale. Registration with the MHRA does not imply that the MHRA agrees with that classification nor even that the classification has been checked by the regulatory body. I'd suggest a tweak along the following lines: "… five (SC1 …) were registered with the MHRA by the manufacturer as Class I medical devices, the classification for the remaining symptom checkers being unclear." I would also question whether this sentence is true any more. One major SC on the market is Ada Health, assuming that is in the included list, Ada's AI product has Class IIa CE marking since December 2022.
228-229	The reference to training data perhaps implies that the diagnostic engine of these symptom checkers is an AI model. Assuming that Babylon is one of the SCs included, reportedly, according to a former employee, the patient pathways are actually hand coded decision trees, the only usage of AI being to interpret patient prompts. Accordingly, the authors may wish to broaden out this statement to also include how these hand coded pathways are coded or perhaps highlight that not all SCs necessarily use AI for their diagnostic engine. Perhaps the authors may also wish to add another point with respect to identifying what model or if AI is even used in SCs given this comment.
231-232	It may be helpful to signpost further discussion of this occurs later in the paper to assist readers.
304-305	As noted above, suggested that there be a slight tweak here: "In this study, most symptom checkers were registered by the manufacturer as a Class I medical device in the UK and were therefore only subject to self-certification prior to roll-out."
310-313	One thing to question here: do the authors wish to claim that triaging is equivalent to a diagnosis here? I'd consider that triaging is perhaps, at best, an indicative diagnosis. I'd consider whether it's more accurate to say something like: "… these systems clearly provide users with information that is relevant to managing disease or injury, behavioural recommendations, and can be viewed as providing an 'indicative diagnosis' in the context of lay usage. Software that provides 'decisive information for making or allowing diagnosis' is sufficient for the device to be at least Class IIa under the UK Medical Device Regulations 2002 according to MHRA guidance." As highlighted in the suggestion, the authors should also specify what regulation the classification is against, as it can vary.
313-316	The authors may wish to also consider whether regulatory scrutiny should also include public understanding of the intended purpose of these devices as this will inform how lay users use or do not use SCs as another numbered item.
316-318	This sentence seems a little misleading. Class IIa and above devices are subject to independent clinical review via Notified / Approved Bodies in both the EU and UK. An appropriately qualified and independent clinician from that Body will review the Clinical Evaluation Report. Additionally, the extent of the FDA review depends upon the pathway used. For instance, most SaMD is 510(k) cleared, meaning the FDA review primarily focuses on whether there is a valid predicate (a similar) device rather than intensive review of clinical evidence. I'd suggest authors rephrase along the following lines: "This could all be aided by independent expert review of these systems, which will occur where the software qualifies as Class IIa and above (in the EU or UK) and where certain FDA pathways are used."
319-320	Many of the previous paragraphs establish that qualification as a medical device alone does not

Confidential

	provide sufficient oversight, the classification is also important. Accordingly, consider the following tweak: "Finally, ensuring that symptom checkers qualify as medical devices and are appropriately classified would allow…"
326-328	This sentence is AI-centric, as previously noted, it seems some of the SCs likely assessed do not actually used AI for their diagnostic engine. The authors may wish to expand to include diversity of decision trees that are hand coded or make this statement explicitly specific to those that use AI as their diagnostic engine.
337-339	Assuming that major SCs such as Babylon and Ada were included, it is matter of public record that these companies had clinical input, whether they listen or listened to this input is perhaps another matter but the authors may wish to ensure that these claims aren't necessarily taken as criticism of the SCs that were reviewed.
339-340	I'm not sure what this sentence means. If it requests manufacturers to make weaker claims with respect to their SC, this will likely reduce the classification under medical device regulation, reducing the probability that there would be independent clinical evaluation.
384	Much of the discussion relates to the regulation of SCs, it seems like a sentence highlighting the high-level recommendation or findings on this matter should be included in the conclusion.

I hope this review and comments are of assistance.

Best regards,

Johan Ordish

Head of Digital Health and Innovation Policy, Roche Diagnostics

Honorary Associate Professor, University of Birmingham

By-Fellow, Hughes Hall, University of Cambridge

Confidential
