# Supplementary material for: Evaluating the diagnostic and triage performance of digital and online symptom checkers for the presentation of myocardial infarction; A retrospective cross-sectional study
Source: PLOS Digit Health. 2024 Aug 5;3(8):e0000558. doi: 10.1371/journal.pdig.0000558 (PMC11299816; doi:10.1371/journal.pdig.0000558)
Supplement: Attachment — Submitted filename: Response to reviewers.docx [file pdig.0000558.s004.docx]

**Regarding the journal requirements:**

1. Please send a completed 'Competing Interests' statement, including any COIs declared by your co-authors. If you have no competing interests to declare, please state "The authors have declared that no competing interests exist". Otherwise please declare all competing interests beginning with the statement "I have read the journal's policy and the authors of this manuscript have the following competing interests:"

**Response**: Thank you – I have updated the Competed Interests statement at the end of our manuscript to these requirements.

2. Please provide separate figure files in .tif or .eps format only and remove any figures embedded in your manuscript file. Please also ensure that all files are under our size limit of 10MB.

**Response**: Thank you – I have provided separate figures in .tif format. Captions including supplementary data captions are provided at the bottom of the main manuscript.

3. In the online submission form, you indicated that "Supporting data may be available upon reasonable request from the corresponding author". All PLOS journals now require all data underlying the findings described in their manuscript to be freely available to other researchers, either 1. In a public repository, 2. Within the manuscript itself, or 3. Uploaded as supplementary information.

This policy applies to all data except where public deposition would breach compliance with the protocol approved by your research ethics board. If your data cannot be made publicly available for ethical or legal reasons (e.g., public availability would compromise patient privacy), please explain your reasons by return email and your exemption request will be escalated to the editor for approval. Your exemption request will be handled independently and will not hold up the peer review process, but will need to be resolved should your manuscript be accepted for publication. One of the Editorial team will then be in touch if there are any issues.

**Response**: Thank you – as per your further correspondence and after consultation with my senior author, I have uploaded our anonymous patient vignettes onto a pubic repository (10.6084/m9.figshare.25872310).

Thank you for bringing this to our attention. I have amended our data availability statement to reflect the above. More than happy to speak to the editorial team if there are any issues.

**Reviewer 1:**

**Response:** Thank you for your comments. Since receiving feedback this reviewer and the second reviewer it has been deemed that a more appropriate term for “accuracy” was “sensitivity” or “performance” due to all the cases having been confirmed to have an MI and so the symptom checkers were being assessed against a set target of 100%. Additionally, the term diagnosis is used by some of the symptom checkers assessed; others may use similar terms, such as “potential conditions causing symptoms”. The latter phrase it is still suggesting a range of differential diagnoses and thus for consistency the term diagnosis was used throughout.

We do respectfully disagree with the reviewer however, that lay people would not view the output as a “diagnosis” given the innate nature of these symptom checkers apps with the users trying to find a reason for their symptoms. Therefore, part of the discussion was regarding to the sensitivity of the diagnostic outputs. As the reviewer kindly points out, triaging information is ultimately more important than diagnosis information in these devices. To maintain patient safety, these devices must be able to offer people advice on when to seek medical care when needed (triaging advice), this formed the original basis of this study as we realised there was potential issues with this.

**Response**: Thank you for your comment. We appreciate that the anonymisation of the included symptom checkers will diminish the impact of our findings and impair the ability of other researchers to replicate our work. However, we made an executive decision to anonymise our findings despite this. Given the high commercial and regulatory interest in this field, any discrepancy between our work and replications may lead to legal exposure. This is especially important given our cross-sectional methodology, where our results were captured at a specific timeframe and may not be replicable given the rapid pace of software development and iteration.

**Response:** Thank you very much for these comments. The point the reviewer raised regarding Painter (2022) is a relevant as this may enhance future study design. However, the data collection, analysis and original manuscript draft was conducted some time ago when there was no such formal guidance existed (partially due to a multiple long review timelines). Thus, a methodology similar to that pre-existing studies was used to assess performance of symptom checkers (Semigran 2015 https://doi.org/10.1136/bmj.h3480).

Second, the reviewer’s point regarding the delay in the writing of the original manuscript and the submission to your paper. This delay was due to multiple factors including multiple long review timelines. We do appreciate the reviewer’s argument, given medical technology is a rapidly ever-developing area of medicine and thus findings in this area of medicine change at an alarming rate, and so the results may vary if we were to re-assess the symptoms checkers at present.

We accept that the ever-changing nature of this topic is a limitation and thus have now also addressed this in the limitations:

“Fifth, the data collection and statistical analysis was conducted during mid-2021. Given this area of medical technology is ever-developing, it is likely that these symptoms checkers have received updates since the initial data collection. Since the data collection, the MHRA classification of the symptom checkers has or is likely to have changed and thus might not represent the current performance of these devices.”

Specific comments:

**Response**: Thank you for your comment. I have amended the section as per your kind suggestion.

“For example, following International Medical Device Regulators Forum (IMDRF) guidance, software that has a medical purpose (as defined in the guidance) qualifies as a medical device and is regulated as such. Despite this apparent international harmonisation, there is still heterogeneity between IMDRF members as to what software qualifies from jurisdiction to jurisdiction.”

**Response**: Thank you for your comment. I have amended this section as per your kind suggestions.

“Although there are some NICE recommended apps such as Sleepio, the majority are not registered as medical devices (Class I, II, etc under CE/UKCA/FDA), nor do they state the classification where they do qualify as a medical device. There is therefore a lack of regulatory requirements relating to safety, such as post-market surveillance requirements.”

**Response**: Thank you for your comment. I have added the below sentence as per your kind suggestion to flat the discussion of symptom checker intended purpose:

“This included seven symptom checkers which could both diagnose and triage patients and one (SC4) that provided only diagnostic information. All the included symptom checkers had disclaimers in their terms of use that they were “not designed to provide medical diagnosis, advice, or treatment”.”

**Response:** Thank you for this helpful suggestion. We have now changed that sentence to:

“At the time of data collection, five of the seven symptom checkers (SC1, SC2, SC3, SC6 and SC7) were registered with the MHRA by the manufacturer as Class I medical devices, the classification for the remaining symptom checkers were unclear.”

The reviewer also points out MHRA class change, as previously mentioned. This has now been addressed in the limitations:

“Fifth, the data collection and statistical analysis was conducted during mid-2021. Given this area of medical technology is ever-developing, it is likely that these symptoms checkers have received updates since the initial data collection. Since the data collection, the MHRA classification of the symptom checkers has or is likely to have changed and thus might not represent the current performance of these devices.”

**Response**: Thank you for your comment. The reviewer is correct in pointing out that not all symptom checkers use AI as part of their decision-making processes and it is further unclear what role, if any, AI plays in any symptom checker that advertises use of AI models. I have amended this section as per your kind suggestions.

“Several included symptom checkers claim to house “AI algorithms” as part of their diagnostic processes. AI-related factors that negatively impact performance include (1) training data that are limited (without the ‘4 Vs’ of big data – volume, variety, velocity and veracity), (2) algorithms with limited accuracy (due to poor choice of predictive factors and poor external validity), (3) a poor user interface that prevents users from navigating the software to best utilise features, and (4) inappropriate intended use of the software. These performance factors represent potential further avenues of development for health technologists and developers (further discussed in this paper’s Recommendations). However, it is currently unclear in what capacity AI is used in the decision-making process of the included symptom checkers, if at all. Indeed, symptom checkers have been reported to use hand-coded decision trees, using AI only for natural language processing to help interpret patient prompts [30,31]. Full transparency is needed in identifying the exact role of AI, if any, in these acutely patient-facing applications.”

**Response**: Thank you for your comment. We have added a sentence to signpost readers to the recommendations made later in the discussion.

“These performance factors represent potential further avenues of development for health technologists and developers (further discussed in this paper’s Recommendations).”

**Response**: Thank you for your comment. I have adjusted the sentence as per your kind suggestion.

“Class I medical devices are self-registered by their manufacture and are therefore only subject to self-certification prior to roll-out.”

**Response**: Thank you for your comment. The reviewer correctly points out that it is important to specify what classification under what regulation symptom checkers are classed as. I have amended the section as per your kind suggestion.

“Despite disclaimers, these systems clearly provide users with information that is relevant to managing disease or injury, behavioural recommendations, and can be viewed as providing an “indicative diagnosis” in the context of lay usage. Software that provides “decisive information for making or allowing diagnosis” is sufficient for the device to be at least Class IIa under the UK Medical Device Regulations 2002 according to MHRA guidance [49].”

**Response**: Thank you for your comment. I agree with the reviewer that evaluation of public understanding of software intended purpose is crucial. I have included this suggestion as a further numbered item.

“Regulatory scrutiny should encompass the entire development pathway, from (1) the datasets used to train and validate the systems, (2) the clinical context in which evaluations were undertaken, (3) reporting of diagnostic and triage performance in the intended clinical setting of use, and (4) evaluating public understanding of the intended purpose of these systems (as this will inform how lay users utilise symptom checkers).”

**Response**: Thank you for your comment and apologies for the misleading statement. I have amended the sentence as per your kind suggestion.

“This could all be aided by independent expert review of these systems, which will occure where the software qualifies as Class IIa and above (in the EU or UK) and where certain FDA pathways are used.”

**Response**: Thank you for your comment. I have amended this sentence as per your kind suggestion to stress the importance of appropriate classification.

“Finally, ensuring that symptom checkers qualify as medical devices and are appropriately classified would allow greater post roll-out scrutiny, which will supersede the current status quo of anecdotal reports through media sources.”

**Response**: Thank you for your comment. I apologise for the confusion. I have changed this sentence to reflect that it refers explicitly to symptom checkers that use AI.

“First, development of symptom checkers that utilise an AI model in their decision-making processes should ideally incorporate real-world patient data.”

**Response**: Thank you for your comment. I have removed this recommendation, given that development teams already contain clinicians who provide clinical context.

**Response**: Thank you for your comment. I have removed this recommendation, as per your kind input – weaker claims will reduce classification and weaken independent clinical evaluation.

**Response**: Thank you for your comment. I have re-written the conclusion to reflect our high-level recommendations regarding regulation.

“This study evaluated the accuracy of eight symptom checkers for diagnosing and triaging myocardial infarctions. Symptom checkers generally provided low accuracy for diagnosing MI. Triage accuracy was higher than diagnostic accuracy, although approximately 20% of cases were under-triaged. Patients who presented with atypical symptoms were under-diagnosed and under-triaged; especially those that were female. Our findings emphasise the need for urgent reassessment of regulation of symptom checkers from policy makers and regulators to ensure good clinical performance, whilst maintaining patient equity and technology transparency.”

**Reviewer 2:**

1. Throughout the paper, the performance of the symptom checkers is evaluated as "accuracy". However, as the population in this study was limited to patients with MI, "sensitivity" would be a more appropriate term to use as a metric of the checkers' performance.

**Response**: Thank you for your comment. The reviewer correctly points out that our study population was limited to only patients with a confirmed myocardial infarction. As such, sensitivity, which measures the proportion of actual positives (diseased subjects) which are correctly identified may be more appropriate. I have changed the text to reflect this – changing accuracy to ‘performance’ and ‘sensitivity’.

For the same reason,

L224, "This indicates a significantly lower positive predictive value for such a life-threatening condition."

**Response**: Thank you for your comment. I have changed the sentence to reflect sensitivity rather that positive predictive value.

L380, "so while we were able to evaluate the positive predictive value of SCs,"

"sensitivity" would probably be appropriate instead of "positive predictive value".

**Response**: Thank you for your comment. In the context of this sentence, we have changed “positive predictive value” to sensitivity.

2. Similar to #1, in L375, "this selection bias would be expected to overestimate the sensitivity of symptom checkers for detecting MI," should be revised. Because the definition of sensitivity itself is the proportion of people who test positive among all those who actually have the disease.

**Response**: Thank you for your comment. I have removed this sentence, as it is misleading.

3. Please give more detailed information about MI. In particular, it would be important for physician readers whether this study included only acute MI, or other types of MI, such as recent MI or OMI.

Thank you for your response, apologies that we had not made this clear. We have addressed this issue accordingly in the Participants section of the Results with the following addition:

“Of the 100 cases collected 97 were confirmed to have an acute ST-Elevation Myocardial Infarction (STEMI), whilst the remaining 3 were found to have an acute Non ST Elevation Myocardial Infarction (NSTEMI).”

4. In the overall test of accuracy differences between the sexes, it would be better to use the Mantel-Henzel test rather than two-tailed t-tests. Please confirm the statistical analyses with a statistician.

Thank you for the comment. Given that the statistical test is assessing for significance between the means of two data sets (n=8 for diagnostic and n=7 for triage) a t-test was more appropriate than a Mantel-Haenszel test, which would require a single discrete data set for both males and females. The same logic was used for comparing the overall test sensitivity between MI type and thus t-tests were also used.

5. Is there an explanation for the "*" in Table 1? If so, please provide the explanation.

**Response**: Thank you for your comment. Apologies for the omission. I have added a note below the table to explain the asterisk.

“Note: * This symptom checker could not provide alternate diagnoses once a serious heart problem was suspected.”
